# Comparison of the Discrimination Performance of AI Scoring and the Brixia Score in Predicting COVID-19 Severity on Chest X-Ray Imaging: Diagnostic Accuracy Study

**DOI:** 10.2196/46817

**Published:** 2024-03-07

**Authors:** Eric Daniel Tenda, Reyhan Eddy Yunus, Benny Zulkarnaen, Muhammad Reynalzi Yugo, Ceva Wicaksono Pitoyo, Moses Mazmur Asaf, Tiara Nur Islamiyati, Arierta Pujitresnani, Andry Setiadharma, Joshua Henrina, Cleopas Martin Rumende, Vally Wulani, Kuntjoro Harimurti, Aida Lydia, Hamzah Shatri, Pradana Soewondo, Prasandhya Astagiri Yusuf

**Affiliations:** 1 Department of Internal Medicine, Pulmonology and Critical Care Division, Faculty of Medicine Universitas Indonesia, RSUPN Dr. Cipto Mangunkusumo Universitas Indonesia Jakarta Indonesia; 2 Department of Radiology, RSUPN Dr. Cipto Mangunkusumo Universitas Indonesia Jakarta Indonesia; 3 Department of Medical Physiology and Biophysics/ Medical Technology Cluster IMERI, Faculty of Medicine Universitas Indonesia Jakarta Indonesia; 4 Department of Internal Medicine, Geriatric Division, Faculty of Medicine Universitas Indonesia, RSUPN Dr. Cipto Mangunkusumo Universitas Indonesia Jakarta Indonesia; 5 Department of Internal Medicine, Nephrology and Hypertension Division, Faculty of Medicine Universitas Indonesia, RSUPN Dr. Cipto Mangunkusumo Universitas Indonesia Jakarta Indonesia; 6 Department of Internal Medicine, Psychosomatic Division, Faculty of Medicine Universitas Indonesia, RSUPN Dr. Cipto Mangunkusumo Universitas Indonesia Jakarta Indonesia; 7 Department of Internal Medicine, Endocrinology – Metabolism – Diabetes division, Faculty of Medicine Universitas Indonesia, RSUPN Dr. Cipto Mangunkusumo Universitas Indonesia Jakarta Indonesia

**Keywords:** artificial intelligence, Brixia, chest x-ray, COVID-19, CAD4COVID, pneumonia, radiograph, artificial intelligence scoring system, AI scoring system, prediction, disease severity

## Abstract

**Background:**

The artificial intelligence (AI) analysis of chest x-rays can increase the precision of binary COVID-19 diagnosis. However, it is unknown if AI-based chest x-rays can predict who will develop severe COVID-19, especially in low- and middle-income countries.

**Objective:**

The study aims to compare the performance of human radiologist Brixia scores versus 2 AI scoring systems in predicting the severity of COVID-19 pneumonia.

**Methods:**

We performed a cross-sectional study of 300 patients suspected with and with confirmed COVID-19 infection in Jakarta, Indonesia. A total of 2 AI scores were generated using CAD4COVID x-ray software.

**Results:**

The AI probability score had slightly lower discrimination (area under the curve [AUC] 0.787, 95% CI 0.722-0.852). The AI score for the affected lung area (AUC 0.857, 95% CI 0.809-0.905) was almost as good as the human Brixia score (AUC 0.863, 95% CI 0.818-0.908).

**Conclusions:**

The AI score for the affected lung area and the human radiologist Brixia score had similar and good discrimination performance in predicting COVID-19 severity. Our study demonstrated that using AI-based diagnostic tools is possible, even in low-resource settings. However, before it is widely adopted in daily practice, more studies with a larger scale and that are prospective in nature are needed to confirm our findings.

## Introduction

### Background

Humans have been learning to adapt to the COVID-19 pandemic. While vaccine development has mitigated the spread, mortality, and morbidity associated with COVID-19, waves of COVID-19 cases are still reported. The main driver of these cases is viral mutation, with the latest mutant, the XBB omicron subvariant, reported to be more virulent and responsible for another COVID-19 wave in Singapore [1]. As of November 1, 2022, a total of 4707 new cases have been reported throughout Indonesia [2]. This is the highest number of new cases reported since September 1, 2022, and might be attributed to SARS-CoV-2 variants. Importantly, at the time this study was conducted, the number of new daily cases reached 14,518 confirmed cases per day at its peak (January 30, 2021) [3].

Currently, the World Health Organization (WHO) endorses the use of the nucleic acid amplification test, including reverse transcription–polymerase chain reaction (RT-PCR), as the gold-standard diagnostic method for COVID-19 cases [4]. Nonetheless, at the height of the pandemic, the weaknesses associated with the test are accentuated, thus increasing the false-negative rates [5,6]. This is similar to the situation we experienced in Indonesia during our study period.

Therefore, another alternative is needed to help with the COVID-19 triage process. Imaging modalities, primarily chest computed tomography (CT) scan and chest x-ray (CXR), are widely available in most health care facilities. Lung CT is the most effective and sensitive method for diagnosing lung lesions during early disease progression [7]. While CXR is less sensitive than lung CT, it is easier to perform, is more cost-effective, is faster, is more portable, has less radiation exposure, has simpler decontamination, and is more widely distributed. Hence, the latter is the initial radiographic modality of choice amid the COVID-19 pandemic [8].

Several scoring systems have been developed to increase CXR’s diagnostic accuracy and reliability in diagnosing COVID-19. The Brixia scoring system (BSS), one of the most commonly used scoring systems, is a semiquantitative CXR scoring system for COVID-19 diagnosis [9]. However, this system is complicated because of the issue of ground truth, which is influenced by rater experience, interobserver agreement, CXR quality, the facility, and the environment surrounding the scoring process. Moreover, additional work burden is imposed on the radiologists as the system relied on manual, subjective scoring and became a less-interesting option at the height of the pandemic [10].

Fortunately, artificial intelligence (AI) is available to ease the workforce burden amid the COVID-19 pandemic. In the past decade, AI has been advancing rapidly, especially in radiology, with applications mainly to diagnose respiratory diseases such as tuberculosis [11]. With the help of AI and machine learning, diagnostic precision can be optimized through computing algorithms for image identification and analysis, resulting in quantitative image scoring [12]. Another important AI role is to determine COVID-19 severity [13], especially in the setting of limited medical resources, equipment, and hospital beds. The correct identification of disease severity can facilitate efficient, adequate, and prompt treatment for those who need it the most.

One of the most used AI software in the COVID-19 pandemic is CAD4COVID x-ray, which detects and scores COVID-19 pneumonia through the color heat map method. This software has been shown to be significantly superior in diagnosing COVID-19 pneumonia through CXR in 454 participants compared with 6 radiologists with an excellent area under the curve (AUC) [14]. However, studies that examined this software utility for disease severity classification remained scarce, especially in low- and middle-income countries such as Indonesia.

Similar to the study mentioned earlier, our study aimed to compare AI performance against that of radiologists. However, in our study, the radiologists used the BSS.

### Objectives

The research questions are two-fold: (1) How does the AI scoring system, using the color heat map methodology, compare to the BSS when assessing CXR in correlation with SARS-CoV-2 RT-PCR results among participants suspected of having COVID-19 pneumonia? (2) What is the effectiveness of the AI scoring system in comparison with the BSS for classifying disease severity in participants suspected of having COVID-19 pneumonia?

The rationale for our research questions is in alignment with the WHO’s guidelines, which recommend the use of chest imaging for the diagnostic evaluation of COVID-19 in scenarios where (1) RT-PCR testing is available but results are delayed or in cases where (2) initial RT-PCR testing returns negative results but there is a strong clinical suspicion of COVID-19 [15]. Our practical experience indicates that these delays in RT-PCR results can extend up to a maximum of 2 weeks. In addition, during periods of high COVID-19 prevalence, the occurrence of false negatives in RT-PCR tests can be notably elevated. A meta-analysis revealed that under conditions of a 50% disease prevalence rate, the rate of misdiagnosis reached 290 out of 1000 participants [16].

Moreover, the wait for a positive RT-PCR result can significantly disrupt the triage system and the clinical flow for patients suspected with COVID-19 infection, consequently leading to delays in the allocation of appropriate treatments. To the best of our knowledge, this is the first study to examine these questions in an Indonesian population.

## Methods

### Study Design

This retrospective cross-sectional diagnostic study used secondary data from medical records and picture archiving and communication system chest radiography repositories. This study was conducted at the Rumah sakit Dr. Cipto Mangunkusumo (RSCM) National Referral Hospital, Jakarta. This study included adults (aged ≥18 years) hospitalized with suspected COVID-19 and RT-PCR–confirmed COVID-19 infection, with or without comorbidities, from April 2020 to April 2021. This study excluded cases with substandard chest radiography qualities, large lung cavities on CXR, concurrent mediastinal or lung mass, and an interval between RT-PCR and CXR acquisition of >7 days. Data were extracted from inpatient medical records from the RSCM department of internal medicine from April 2020 to April 2021 that met the inclusion criteria. Sampling was performed consecutively until the minimum number of samples was obtained.

### Ethical Considerations

This study protocol was reviewed and approved by the Faculty of Medicine, University of Indonesia’s Ethical Board (approval number Nomor KET-588/UN2. F1/ETIK/PPM.00.02/2020). Written informed consent was waived because of the retrospective nature of the study and amid the COVID-19 pandemic. All medical records and CXRs were deidentified and anonymized to ensure patient confidentiality and compliance with privacy standards. No compensations were given to the participants because of the nature of the study.

### Operational Definition

Vaccination data were ascertained from history taking and medical records and ordinally stratified into not vaccinated, vaccinated once, and vaccinated twice. COVID-19 disease severity was determined on hospital admission by emergency medical doctors and was stratified according to the local Indonesian guideline, which adopted the WHO COVID-19 disease severity stratification (Table S1 in Multimedia Appendix 1) [17]. Oxygen saturation data were measured using the transmittance pulse oximeter. They were ordinally stratified according to normal oxygen saturation (94%-100% on room air), mild to moderate hypoxia (90%-93% on room air), and severe hypoxia (<90% on room air). The RT-PCR data were ascertained from the medical records and used naso-oropharyngeal specimens. Specimen handling and processing for RT-PCR have been described elsewhere [18].

Diabetes was defined according to the American Diabetes Association and the Indonesian Guidelines for the Management and Prevention of Diabetes [19]. Hypertension was defined according to the Indonesian Society of Hypertension Guidelines and the Eighth Joint National Committee [20,21]. Chronic obstructive pulmonary disease and asthma were determined according to the Global Initiative for Obstructive Lung Disease and the Global Initiative for Asthma guidelines, respectively [22,23]. Finally, pulmonary tuberculosis was defined according to the WHO guideline. It was deemed positive if there was a previous history of tuberculosis or active pulmonary tuberculosis [24]. Acute respiratory distress syndrome was defined according to the Berlin criteria [25].

### BSS Measurement

The BSS is a semiquantitative method used to measure the severity of lung lesions on CXR. The method has been described in detail elsewhere [9]. Briefly, the lung image on CXR is divided into 6 zones, and each zone can have a score of 0 to 3 with a total maximum score of 18. A total of 2 observers, both board-certified radiologists, measured the score. The third radiologist acted as the ground truth, with >20 years of experience. Every CXR was anonymized before the scoring was performed, and radiologists were blinded to the clinical data. In this study, we focused on the overall CXR score domain of BSS for comparison. We did not establish a predefined BSS cutoff for the classification of positive SARS-CoV-2 RT-PCR results and for the disease severity classification in advance.

### AI System for CXR Interpretation

For AI-based CXR interpretation, we used the CAD4COVID x-ray software. It is an advanced AI system built upon deep learning techniques designed for the identification of COVID-19 indicators in frontal CXRs. This cutting-edge system, an extension of the commercially available CAD4TB software (version 6; Thirona), primarily developed for tuberculosis detection in chest radiographs, undergoes initial preprocessing steps, including image normalization and lung segmentation via U-net software. Subsequently, the system uses a patch-based analysis with a convolutional neural network and concludes with image-level classification using an ensemble of networks [14].

The following steps were performed on the software:

1. Digital CXR (Digital Imaging and Communications in Medicine) of patients suspected with COVID-19 infection was pseudoanonymized before image upload with the picture archiving and communication system INFINITT software (INFINITT Healthcare).

2. CXR scoring was performed in 4 consecutive steps, which are as follows:

- Normalization: this step was to normalize the CXR scale from CXRs with larger or smaller sizes and to be generalized by AI so that it can be processed uniformly.
- Lung fields segmentation: the AI automatically did this step to delineate the lungs and distinguish them from the rest of the image.
- Texture analysis: this step was to determine relevant abnormalities in lung segments.
- Finally, area analysis was done to estimate the percentage of involved lung parenchyma.

3. All filter weights were calculated. The average filter weight was used as a mask on the CXR image to generate a color heat map, which was visualized only in the lung area previously segmented by the previously trained model.

4. The color heat map produced different colors corresponding to its weight. Red, yellow, green, and blue correspond to high, medium, low, and very low probability of abnormality on the CXR, respectively.

5. The digital CXR was uploaded to the CAD4COVID software to generate 2 AI scorings. First, the affected lung area (ALA) score, with a scale from 0 to 100, was determined according to the total lung volume with abnormalities detected on the CXR. A higher value indicates more lung area that is affected. Second, the COVID-19 probability score, with a scale from 0 to 100, was determined according to the average final weight of all layers. A higher value indicates a higher probability of COVID-19.

The CAD4COVID cutoff for a positive SARS-CoV-2 RT-PCR result and disease severity classification were determined during the study.

### Data Analysis

#### Descriptive Statistics

We summarized baseline characteristics, presenting categorical variables as frequencies (n) and proportions (%). Continuous variables were described as means with SDs for normally distributed data and as medians with IQRs for nonnormally distributed data. The normality of continuous data was assessed using the Kolmogorov-Smirnov test.

#### Interobserver Reliability

Interobserver reliability was evaluated using the intraclass correlation coefficient (ICC) with a 2-way mixed-effect model (*k*=2) and consistency. We categorized ICC values as poor, moderate, good, or excellent reliability. Estimated means with their respective 95% CIs were reported for each respective ICC. ICC values of <0.50, 0.50 to 0.75, 0.75 to 0.90, and >0.90 were interpreted as poor, moderate, good, and excellent reliability, respectively. In addition, we also evaluated interobserver reliability based on CXR projections, that is, posteroanterior (PA) and anteroposterior (AP).

#### Receiver Operating Characteristic Analysis

To assess the diagnostic performance of both AI scoring and Brixia scoring, we used receiver operating characteristic curves and AUC analyses. AUC values of <0.60, 0.60 to 0.70, 0.70 to 0.80, 0.80 to 0.90, and 0.90 to 1 were classified as failure, poor, fair, good, and excellent, respectively. The results were calibrated and internally validated using the Hosmer-Lemeshow test and bootstrapping. A comparison between the AUCs was performed using the DeLong test. A *P* value of <.05 indicates significant difference.

#### Optimum Cutoff Values

We determined the optimum cutoff values for AI scoring (probability score and affected lung score) and the BSS to distinguish the RT-PCR results and classify disease severity. The Youden Index method guided our selection process, aiming for the highest sensitivity with a specificity of ≥50% (Tables S2 to S4 in Multimedia Appendix 1).

#### Diagnostic Performance

We calculated sensitivity, specificity, accuracy, positive predictive value, and negative predictive value based on the chosen cutoff values. The reference standard for COVID-19 diagnosis was RT-PCR, as it is the diagnostic modality needed to confirm COVID-19. The reference standard for disease severity classification was the BSS.

#### Agreement Tests

The agreement between AI and Brixia scoring in relation to RT-PCR results and disease severity was assessed using the kappa statistic. Kappa values of 0 to 0.20, 0.21 to 0.40, 0.41 to 0.60, 0.61 to 0.80, and 0.81 to 1 were classified as slight, fair, moderate, substantial, and near-perfect agreement, respectively.

We adhered to the Standards for Reporting of Diagnostic Accuracy statement in our reporting of results. Statistical analyses were performed using SPSS for Macintosh (version 27; IBM Corp), MedCalc for Windows (version 20.114; MedCalc Software Ltd), and Stata Statistical Software for Macintosh (version 14; StataCorp LP)

## Results

**Figure 1:**
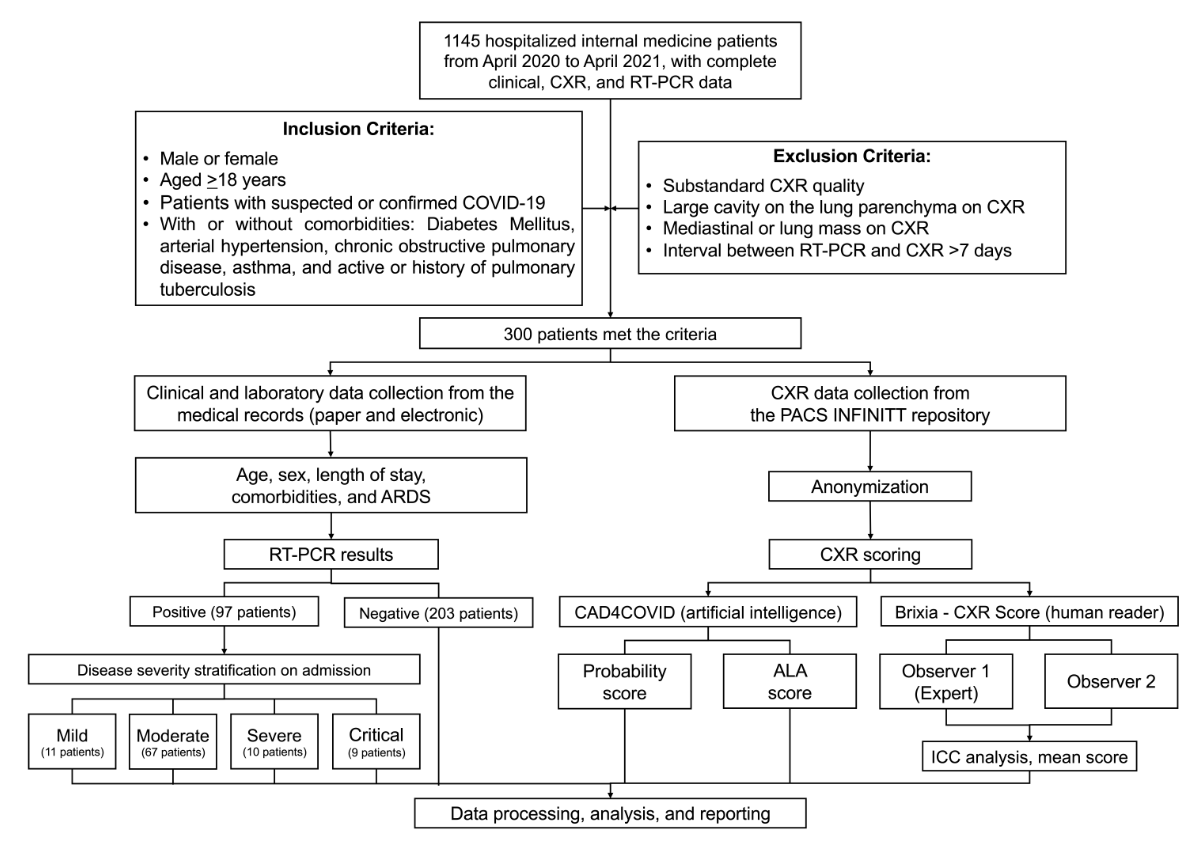
Flowchart depicting the study outline. ALA: affected lung area; ARDS: acute respiratory distress syndrome; CXR: chest x-ray; ICC: intraclass correlation coefficient; PACS: picture archiving and communication system; RT-PCR: reverse transcription–polymerase chain reaction.

From April 2020 to April 2021, there were 1145 hospitalized patients with COVID-19 in RSCM National Referral Hospital, Jakarta, with complete clinical data, CXR, and RT-PCR. Only 26.2% (300/1145) of the participants met the inclusion and exclusion criteria. The study outline is presented in Figure 1.

### Baseline Characteristics

A total of 300 participants (refer to the Methods section) were enrolled in this study. Demographics, comorbidities, clinical data, and radiology scoring are presented in Table 1.

In this study, most hospitalized patients with COVID-19 were aged <60 years (211/300, 70.3%), with a median population age of 52 (IQR 39.0-61.0) years and male gender preponderance (159/300, 53%). In total, >two-thirds of the patients had a negative RT-PCR result (203/300, 67.7%). Moderate disease severity dominated COVID-19 disease severity. Moreover, the 3 most common comorbidities were hypertension, diabetes, and pulmonary tuberculosis.

**Table 1:** Baseline characteristics of study participants (N=300).

Variables			Values
Age (years), median (IQR^a^)			52.0 (39.0-61.0)
**Age (years), n (%)**			
	>60	89 (29.7)	
	<60	211 (70.3)	
**Sex, n (%)**			
	Male	159 (53)	
	Female	141 (47)	
**Oxygen saturation^b^, n (%)**			
	Normal	132 (44)	
	Mild to moderate hypoxia	18 (6)	
	Severe hypoxia	25 (8.3)	
**RT-PCR^c^, n (%)**			
	Positive	97 (32.3)	
	Negative	203 (67.7)	
**Disease severity, n (%)**			
	Mild	42 (14)	
	Moderate	178 (59.3)	
	Severe	27 (9)	
	Critical	53 (17.7)	
**Comorbidities, n (%)**			
	Diabetes mellitus	77 (25.7)	
	Hypertension	96 (32)	
	COPD^d^	2 (0.7)	
	Asthma	4 (1.3)	
	Pulmonary tuberculosis	16 (5.3)	
ARDS^e^, n (%)			45 (15)
Length of stay (days), median (IQR)			8.0 (4.0-13.3)
Overall CXR^f^ score-Brixia score, median (IQR)			3.00 (0.0-9.5)
**AI^g^-CAD4COVID, median (IQR)**			
	Probability score^h^	62.0 (35.75-83.25)	
	ALA^i^ score^j^	6.5 (1.0-27.0)	

^a^Normally distributed data are presented as mean (SD). Otherwise, it is presented as median (IQR).

^b^The sum of participants falls short of 300 since room air peripheral oxygen saturation data was missing for 125 patients.

^c^RT-PCR: reverse transcription–polymerase chain reaction.

^d^COPD: chronic obstructive pulmonary disease.

^e^ARDS: acute respiratory distress syndrome.

^f^CXR: chest x-ray.

^g^AI: artificial intelligence.

^h^Higher AI probability scores are commensurate with a higher COVID-19 probability.

^i^ALA: affected lung area.

^j^Higher AI scores of affected lung area are commensurate with a larger affected lung area.

### Interobserver Reliability of Lung Lesion Severity on CXR With the BSS

The analysis showed no statistically significant difference for every lung zone evaluation and the total score in Brixia scoring between the 2 observers (Table 2).

The ICC score for the BSS for every lung zone was >0.75, with good to excellent reliability for zone A (right upper lobe [RUL]) and zone D (left upper lobe [LUL]). Excellent reliability was noted for zone B (right middle lobe), zone C (right lower lobe), zone E (left middle lobe), zone F (left lower lobe), and for the overall CXR score. The Brixia score for each lung zone and the overall CXR score had similar proportions for both AP (187/300, 62.3%) and PA (101/300, 33.7%) CXR projections. The AP had a lower ICC score with a wider 95% CI (Figure S1 in Multimedia Appendix 1).

**Table 2:** Difference for every lung zone evaluation and the total score in Brixia scoring between the 2 observers.

Scoring parameter	Observer, median (IQR)			*P* value^a^
	1	2		
Zone A (RUL^b^)	0.0 (0.0-1.0)	0.0 (0.0-1.0)	.33	
Zone B (RML^c^)	0.0 (0.0-2.0)	0.0 (0.0-2.0)	.45	
Zone C (RLL^d^)	1.0 (0.0-2.0)	1.0 (0.0-2.0)	.99	
Zone D (LUL^e^)	0.0 (0.0-0.0)	0.0 (0.0-1.0)	.37	
Zone E (LML^f^)	0.0 (0.0-2.0)	0.0 (0.0-2.0)	.61	
Zone F (LLL^g^)	1.0 (0.0-2.0)	1.0 (0.0-2.0)	.45	
Overall CXR^h^ score	3.0 (0.0-9.25)	3.0 (0.0-10.0)	.55	
Δ Overall CXR score	0.0 (0.0-1.0)	N/A^i^	N/A	

^a^*P*<.05 is considered statistically significant.

^b^RUL: right upper lobe.

^c^RML: right middle lobe.

^d^RLL: right lower lobe.

^e^LUL: left upper lobe.

^f^LML: left middle lobe.

^g^LLL: left lower lobe.

^h^CXR: chest x-ray.

^i^N/A: not applicable.

### Performance Comparison Between Color Heat Map–Based AI Scoring Performance and the BSS on CXR Against SARS-CoV-2 RT-PCR Results of Patients Suspected With COVID-19 Infection

Of the 300 participants suspected with COVID-19 infection, only 32.3% (97/300) had a positive RT-PCR result. Owing to the small number of RT-PCR–positive cases and its large measurement error, no scoring system was able to statistically discriminate between patients who had a positive RT-PCR result and those who had a negative RT-PCR result.

### Performance Comparison Between Color Heat Map–Based AI Scoring and the BSS on CXR Against COVID-19 Disease Severity

**Figure 2:**
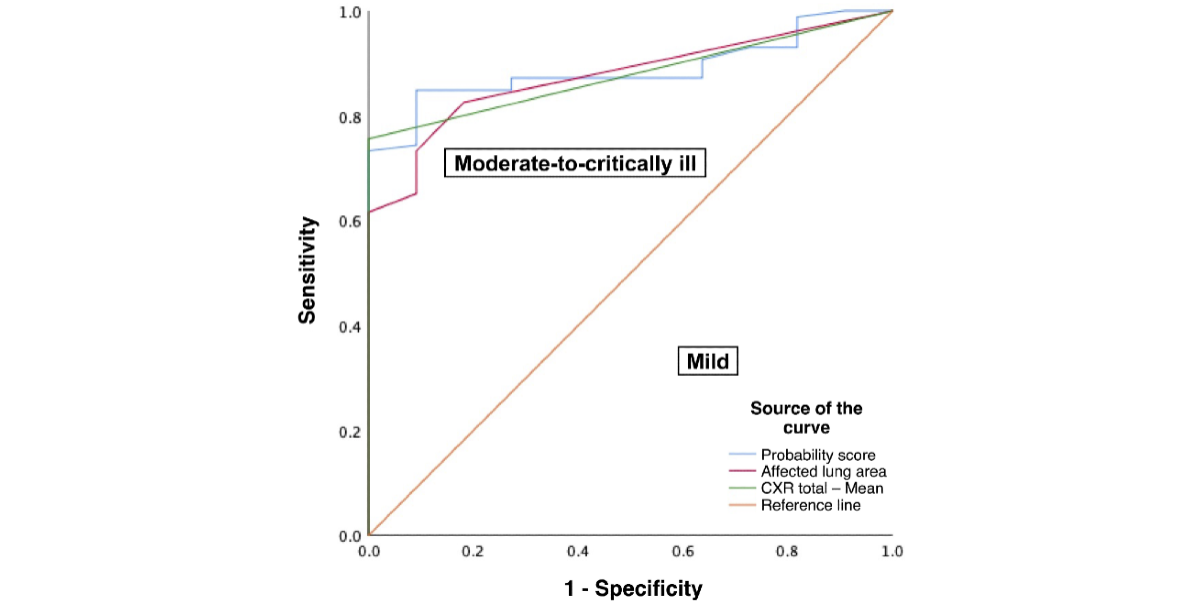
Receiver operating characteristic curves of artificial intelligence and Brixia scoring systems against disease severity of suspected and confirmed patients with COVID-19. CXR: chest x-ray.

All scores were higher among 86% (258/300) of cases of moderate to critical disease, when compared with 14% (42/300) of cases of mild disease (*P*<.001). The receiver operating characteristic analysis showed that the AI probability score, AI ALA score, and BSS had excellent discrimination against COVID-19 disease severity (Table 3; Figure 2).

Compared with the performance of BSS to discriminate disease severity (sensitivity 75.7% and accuracy 79.3%), the AI ALA score had better sensitivity and accuracy (Sn 84.5% and accuracy 83.0%), while the AI probability score did not (Sn 68.2% and accuracy 69.7%). The kappa statistic showed that there were moderate agreements between AI probability score (κ=0.271±0.050; *P*<.001), AI ALA score (κ=0.452±0.063; *P*<.001), and BSS (κ=0.456±0.053; *P*<.001) against COVID-19 disease severity (Table 3). However, there was no significant difference between the AUC for the AI probability score (AUC 0.787) and the BSS (AUC=0.863), with a difference of 0.076 (SD 0.034, 95% CI 0.010-0.142; *P*=.04). Similarly, no significant difference was observed between the AI ALA score (AUC 0.857) and the BSS, with a negligible difference of 0.006 (SD 0.023, 95% CI –0.039 to 0.052; *P*=.76), indicating that both AI scores were comparable to BSS in discriminating disease severity (Table 4).

**Table 3:** AUC^a^, optimum cutoff, Sn^b^, Sp^c^, positive predictive value, negative predictive value, and diagnostic accuracy for AI^d^ scores (probability and ALA^e^) and BSS^f^ in discriminating 86% (258/300) of cases of moderate to critical disease from 14% (42/300) of cases of mild disease^g^.

	AUC, mean (SD)	*P* value^h^	Value, 95% CI	Cutoff	Sn, %	Sp, %	Acc^i^, %	PPV^j^, %	NPV^k^, %
AI probability score	0.787 (0.033)	<.001	0.722-0.852	≥56	68.2	78.6	69.7	95.1	28.7
AI ALA score	0.857 (0.024)	<.001	0.809-0.905	≥1	84.5	73.8	83.0	95.2	43.7
BSS	0.863 (0.023)	<.001	0.818-0.908	≥1	76.7	95.2	79.3	99.0	40.0

^a^AUC: area under the curve.

^b^Sn: sensitivity.

^c^Sp: specificity.

^d^AI: artificial intelligence.

^e^ALA: affected lung area.

^f^BSS: Brixia scoring system.

^g^Interpretation: <0.60: fail; 0.60 to 0.70: poor classification; 0.70 to 0.80: fair classification; 0.80 to 0.90: good classification; 0.9 to 1: excellent classification.

^h^*P*<.05 was considered statistically significant and emphasized by bold texts.

^i^Acc: accuracy.

^j^PPV: positive predictive value.

^k^NPV: negative predictive value.

**Table 4:** The comparison between AI^a^ and Brixia scoring AUC^b^ values against COVID-19 disease severity.

	AUC, mean difference (SD)	95% CI	*P* value^c^
AI probability score vs BSS^d^	0.076 (0.034)	0.010 to 0.142	.04
AI ALA^e^ score vs BSS	0.006 (0.023)	–0.039 to 0.052	.76

^a^AI: artificial intelligence.

^b^AUC: area under the curve.

^c^*P*<.05 is considered statistically significant.

^d^BSS: Brixia scoring system.

^e^ALA: affected lung area.

The distribution of the 2 binarized groups for all 3 scores is illustrated in a histogram (Figure S2 in Multimedia Appendix 1).

The Hosmer-Lemeshow test shows good calibration and internal validation for the AI probability score (*P*=.90) and AI ALA score (*P*=.99). Calibration and internal validation of the BSS could not be performed.

## Discussion

### Principal Findings

In this study, we demonstrated that the BSS had excellent interobserver reliability (ICC>0.75) to determine the severity of lung lesions on CXR (ICC 0.950, 95% CI 0.937-0.960). Our findings are in agreement with previous studies by Mruk et al [26] (ICC=0.847, 95% CI 0.816-0.848) and Chaudhari et al [27] (ICC=0.920, 95% CI 0.880-0.950). Our study also aligns with that of Mruk et al [26], who reported that PA projection had higher interobserver agreement and reliability than AP projection. Thus, it can be assumed that PA CXR projection has better image quality and thus can affect the interpretation.

In the context of lung anatomy, zone A (RUL) and zone D (LUL) had lower ICC values than the other zones. These findings were similar to those of a previous study by Monaco et al [28], who also reported that LUL had the lowest ICC, followed by the right lower lobe and the RUL. A hypothesis explaining these findings is the left upper zone sparing phenomenon seen in COVID-19 pneumonia, that is, infiltrates rarely develop on the LUL and the RUL. Moreover, anatomical structure juxtaposition with the clavicles and scapula influences the scoring subjectivity of these zones.

Although the interobserver reliability was excellent and the mean difference in the overall CXR score was low, the interobserver score had a very wide range (Δ *Overall* CXR *score=*0.0-16.0). This difference was influenced by a myriad of factors that were not studied in this research. Similarly, van Assen et al [29] reported poor interobserver agreement when assessing disease severity. The disease severity classification was different in 82% of CXR, with 59% showing a 1° difference in disease severity. The differences were primarily observed in the intermediate group (mild and moderate severity), which can greatly affect clinical decision-making for patient management.

While the semiquantitative method may be seen as more convenient, the subjective nature of this scoring system can give rise to multiple problems, including how to determine the ground truth. Thus, supporting data is needed to justify its use [10]. Moreover, the BSS can contribute to health care workers’ burnout, especially radiologists, as the system relies on them to manually sum up the score.

No scoring system was able to statistically discriminate between which patients had a positive RT-PCR result and which ones had a negative RT-PCR result. These findings might be explained by the low prevalence of positive RT-PCR results (97/300, 32.3%), which might have been caused by a high false-negative rate, considering the true COVID-19 prevalence during the participant enrollment was 47.6% (143/300). False-negative RT-PCR results can be explained by numerous factors, including exposure time, symptom onset, SARS-CoV-2 virulence, and specimen handling and processing [30-35].

Lai and Lam [31] showed that the interval between the day of exposure and the day of RT-PCR specimen sampling contributed to false-negative rates. False-negative rates of RT-PCR for the 0-, 5-, 8-, and 21-day interval were 100%, 35%, 20%, and 66%, respectively. Viral virulence also contributed to a false-negative rate, as reported in studies by Alteri et al [34] and Petrillo et al [35]. They showed that the false-negative rates of participants infected with low viral virulence were approaching 20% to 30%.

Another possible contributor to an increased false-negative rate was specimen transportation, considering that the viral specimen should be kept at a minimum of –70 °C to maintain viral isolation and viability [36]. This factor is crucial owing to the unavailability of an in-house RT-PCR facility in our hospital because it was centralized in the early days of the COVID-19 pandemic. Our hospital is a national referral hospital, which might have led to referral bias. Therefore, most patients referred from lower-tier health care facilities with COVID-19 may have negative RT-PCR results as they may have passed the virulence period and many days have passed since the first onset of symptoms. Finally, according to the Indonesian COVID-19 guidelines, the RT-PCR test did not have to be repeated, contrary to the WHO and European Center for Disease Control guidelines, which state that before discharging patients, 2 negative RT-PCRs are needed [4,37].

Therefore, in our circumstances, solely relying on RT-PCR can lead to an underestimation of the COVID-19 diagnosis, which will impact the decision-making and clinical management of COVID-19. In retrospect, as the RT-PCR turnaround time is high with a high operating cost, at the height of the COVID-19 pandemic, another screening modality that is fast, inexpensive, practical, reliable, and noninvasive is needed for triaging, diagnosing, and quarantining suspected COVID-19 cases as measures to curb the pandemic. Nonetheless, this modality will act as an adjunct to conventional CXR and should be incorporated while waiting for the RT-PCR results [38]. Therefore, we preferred CXR rather than CT scan as the modality that we researched because it is widely available, inexpensive, and has low radiation exposure.

Although our findings found that AI and Brixia scoring were poor COVID-19 diagnostic modalities, they were similar to previous studies that also reported that AI and manual radiologist scoring had similar performance. Murphy et al [14] showed that the AI system (AUC 0.810) gave similar scoring results and was even superior compared with 6 radiologists (*P*<.001). This was likely because of the heterogeneous lung lesions seen on CXR in patients with COVID-19, including peripheral and diffuse opacities, which made distinguishing COVID-19 from other pulmonary diseases more challenging. In contrast, Chamberlin et al [39] stated that radiologists had a superior diagnostic ability for COVID-19 (AUC 0.936, 95% CI 0.918-0.960) compared to AI (AUC 0.890, 95% CI 0.861-0.920), despite similar discriminatory abilities between them.

However, the AI scoring system has several advantages over manual evaluation for CXR interpretation. First, an automated and quantified AI scoring system will decrease radiologists’ overall work burden. Second, with the help of the AI scoring system, radiologists can increase their accuracy up to 99.05%, which is comparable to that of RT-PCR [40,41]. Moreover, as nonradiologists, especially medical doctors who work on the front line, are the first ones to see the CXR before interpretation by the radiologists, AI use can increase the diagnostic agreement. Hwang et al [42] showed that AI scoring gave similar results to radiologists (AUC 0.714 vs 0.712) but was superior to nonradiologists (AUC 0.714 vs 0.584). Furthermore, hybrid AI use increased diagnostic agreement significantly for both groups (radiologists’ Fleiss κ=0.688, 95% CI 0.665-0.710; nonradiologists’ Fleiss 𝜅=0.510, 95% CI 0.488-0.533) [42].

In our assessment of AI and BSS performance against disease severity in patients suspected with COVID-19 infection participants, the AI ALA score exhibited higher sensitivity and diagnostic accuracy compared with the BSS, although the difference was not statistically significant. This can be explained by the fact that CAD4COVID can eliminate the technical limitations of conventional CXR, such as its quality. Furthermore, before generating scores, the software normalized the CXR image and segmented lung fields, which optimized CXR image quality [14]. Considering the limited size of our data set, it is not possible to definitively assert the superiority of the AI system over human Brixia. Nonetheless, our analysis indicates a 95% CI that the AI system’s performance is not statistically distinct from that of human Brixia. This finding serves as a promising safety signal, warranting additional comprehensive testing and assessment of AI scoring systems in larger subsequent studies aimed at real-world implementation. Regardless, both had excellent discrimination without significant differences in AUC.

In the context of evaluating AI and Brixia scoring in assessing disease severity among patients suspected with COVID-19 infection participants, both AI scoring systems demonstrated notably higher sensitivity and diagnostic accuracy when compared with the BSS. This superiority can be attributed to the capacity of CAD4COVID to overcome the inherent technical limitations often associated with conventional CXR imaging, including variations in image quality. Notably, CAD4COVID effectively normalized CXR images and meticulously segmented lung fields before generating probability and ALA scores, thereby enhancing overall image quality. Notably, both AI and human Brixia scoring exhibited excellent discrimination without significant disparities in the AUC.

To the best of our knowledge, this is the first study to compare the AI scoring performance using the CAD4COVID software based on AI probability and AI ALA score with the BSS against disease severity in patients suspected with COVID-19 infection participants. Our findings align with those of Guiot et al [43], albeit with a different modality. In their research, CAD4COVID-CT, through the ALA and CT severity score (CT-SS), was able to predict the length of stay, the odds of intensive care unit admission, the odds of mechanical ventilation, and the odds of in-hospital mortality [43]. The cutoff value chosen for odds of intensive care unit admission was CT-SS 14 with an AUC of 0.84 (95% CI 0.79-0.90) and, for odds of mechanical ventilation, it was CT-SS 16 with an AUC of 0.71 (95% CI 0.63-0.78).

The sensitivity, specificity, and accuracy of the BSS were 75.6%, 100%, and 78.4%, respectively. These values were lower than reported by Abo-Hedibah et al [44]. In their study, the BSS sensitivity, specificity, and accuracy in diagnosing moderate disease were 90.4%, 100%, and 94.6%, respectively, whereas in diagnosing severe disease, they were 100%, 84.5%, and 86.7%, respectively. Nevertheless, they referred to disease severity stratified by the WHO, whereas our study referred to the Indonesian national guidelines.

The clinical implications of these findings are 2-fold. First, with the help of AI scoring, a clinician can more confidently exclude moderately to critically ill patients with COVID-19. However, the AI scoring system is image based and not clinically applicable. Thus, it will generate conflicting results when the disease severity classification relies on clinical criteria, as seen in the WHO and Indonesian guidelines [17,45]. In contrast, the US National Institutes of Health COVID-19 guidelines do not solely rely on clinical data [46]. For example, moderate COVID-19 infection can be diagnosed if there are pulmonary infiltrates on imaging. As COVID-19 pneumonia can be asymptomatic or without typical signs and symptoms of pneumonia, we argue that disease severity should be stratified according to the National Institutes of Health guideline [46].

Second, minimal pulmonary lesions on the CXR that are sometimes undetected by manual readers could be identified by AI. The AI probability and the ALA score had higher sensitivities than the BSS to rule out moderate to critical disease. The AI probability and the ALA score can mitigate drawbacks when relying on clinical judgment and conventional radiographs. These findings are significant, as the downstream effect will include patient management, that is, outpatient or inpatient treatment.

According to our study results, we propose the incorporation and clinical application of AI use on CXR as an ancillary diagnostic tool for patients suspected with COVID-19 infection in a structured algorithm. We hope that for future COVID-19 outbreaks, this algorithm can shorten triage and diagnostic time and shorten clinical decision-making as to whether the patients need to be quarantined or hospitalized. As AI and Brixia scoring did not have discriminatory ability against RT-PCR results in suspected COVID-19 cases, we hope that with the addition of clinical and laboratory data, a more precise diagnostic model can be developed. In contrast to the RT-PCR results, the AI and Brixia scoring had an excellent ability to discriminate disease severity in patients suspected with COVID-19 infection, with superior sensitivity and accuracy observed for the former. Thus, AI scoring can be considered for CXR interpretation because of the clinical–radiological incompatibility that can sometimes be observed in patients with COVID-19 pneumonia.

**Figure 3:**
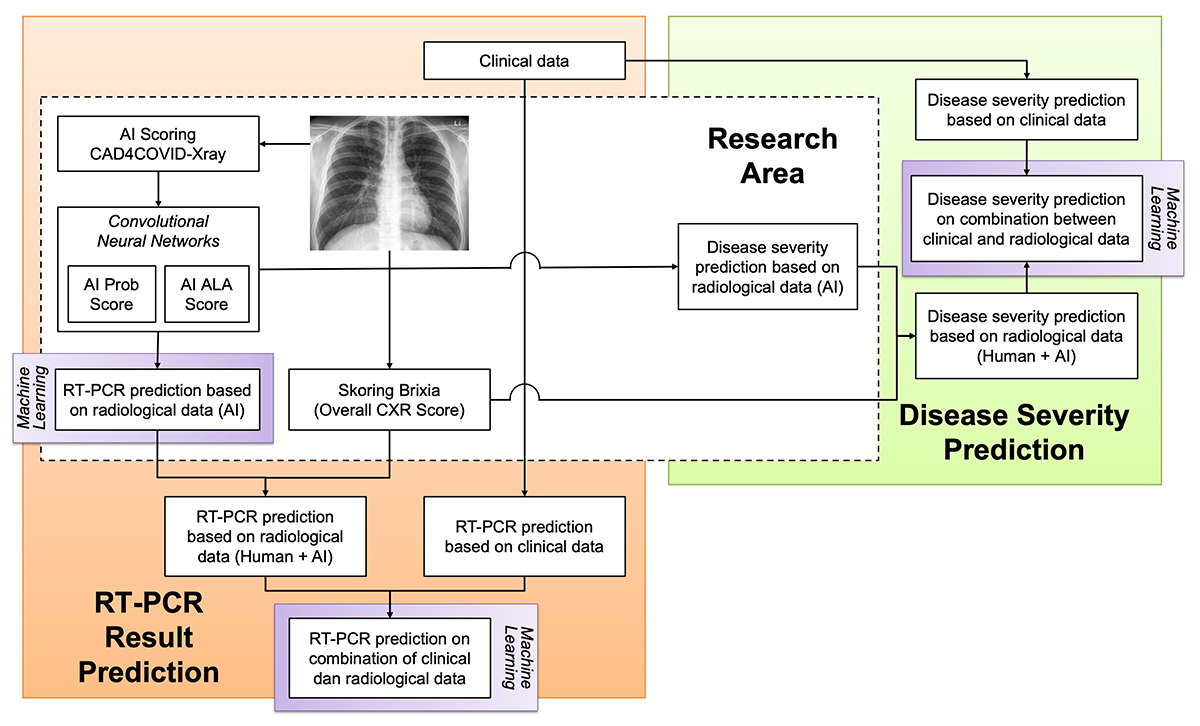
Proposed algorithm of artificial intelligence (AI) incorporation and AI application on chest x-ray as an ancillary diagnostic test on patients with COVID-19. ALA: affected lung area; CXR: chest x-ray; Prob: probability; RT-PCR: reverse transcription–polymerase chain reaction.

Our study showed that the AI scoring system has the potential to become a disease severity classifier for patients with suspected COVID-19 infection. As the AI scoring system was generated through machine learning, with more data available to train the system, the AI scoring accuracy will continue to increase. We proposed an algorithm for AI incorporation and AI application on CXR as an ancillary diagnostic test for patients with COVID-19 (Figure 3).

### Limitations

Our study has several limitations. First, the study population is relatively small compared with the COVID-19 prevalence during the participant’s enrollment, owing to missing data and the study’s exclusion criteria. Second, as our hospital is a national referral hospital, most patients came with moderate to critical disease and presented with other pulmonary lesions. Furthermore, several study variables could not be retrieved as they were not incorporated into the patient’s medical record. Finally, in the early days of the pandemic, our hospital did not have an in-house RT-PCR facility because of the centralized specimen processing, so the specimen had to be delivered to another facility, which could further compromise RT-PCR results.

### Conclusions

The AI score for the ALA and the human radiologist Brixia score had similar and good discrimination performance in predicting COVID-19 severity. Our study demonstrated that using AI-based diagnostic tools is possible, even in low-resource settings. However, before it is widely adopted in daily practice, more studies with a larger scale and prospective in nature are needed to confirm our findings.

## Appendix

Multimedia Appendix 1 Supplementary tables and figures.

## Abbreviations

AI: artificial intelligence
ALA: affected lung area
AP: anteroposterior
AUC: area under the curve
BSS: Brixia scoring system
CT: computed tomography
CT-SS: computed tomography severity score
CXR: chest x-ray
ICC: intraclass correlation coefficient
LUL: left upper lobe
PA: posteroanterior
RSCM: Rumah sakit Dr. Cipto Mangunkusumo
RT-PCR: reverse transcription–polymerase chain reaction
RUL: right upper lobe
WHO: World Health Organization

## Data Availability

The data that support the findings of this study are available from the corresponding author on reasonable request.
