# Supplementary material for: Comparison of the Discrimination Performance of AI Scoring and the Brixia Score in Predicting COVID-19 Severity on Chest X-Ray Imaging: Diagnostic Accuracy Study
Source: JMIR Form Res. 2024 Mar 7;8:e46817. doi: 10.2196/46817 (PMC10958333; doi:10.2196/46817)
Supplement: Multimedia Appendix 1 [file formative_v8i1e46817_app1.docx]

**Supplementary Table 1.** Disease severity classifications according to the Indonesian COVID-19 guideline.

| Disease Severity | Signs and Symptoms |
| --- | --- |
| Asymptomatic | No sign and symptoms |
| Mild | Patients with symptoms but no evidence of viral pneumonia or hypoxia.  Symptoms include fever, cough, fatigue, anorexia, shortness of breath, and myalgia. Other nonspecific symptoms such as sore throat, nasal congestion, headache, diarrhea, nausea and vomiting, loss of smell (anosmia), or loss of taste (ageusia) that appear before the onset of respiratory symptoms are also frequently reported. In older patients and immunocompromised individuals, atypical symptoms such as fatigue, decreased consciousness, decreased mobility, diarrhea, loss of appetite, delirium, and absence of fever are common. |
| Moderate | Patients with clinical signs of pneumonia (fever, cough, shortness of breath, rapid breathing) but no signs of severe pneumonia, including SpO2 > 93% on room air. |
| Severe | Patients with clinical signs of pneumonia (fever, cough, shortness of breath, rapid breathing), plus one of the following: respiratory rate > 30 breaths per minute, severe respiratory distress, or SpO2 < 93% on room air. |
| Critical | Patients with sepsis, septic shock, or acute respiratory distress syndrome (ARDS) |

Source: Indonesian COVID-19 Guideline 4^th^ Edition.

**Supplementary Table 2.** Optimum cut-off value calculation for AI probability score against moderate-to-critical disease.

| Cut point (>) | Sensitivity | Specificity | Accuracy | LR+ | LR- | Youden Index |
| --- | --- | --- | --- | --- | --- | --- |
| 0 | 100.00% | 0.00% | 86.00% | 1.0000 |  | 0.00 |
| 1 | 100.00% | 2.38% | 86.33% | 1.0244 | 0.0000 | 0.02 |
| 2 | 99.22% | 7.14% | 86.33% | 1.0686 | 0.1085 | 0.06 |
| 3 | 97.29% | 9.52% | 85.00% | 1.0753 | 0.2849 | 0.07 |
| 4 | 96.51% | 11.90% | 84.67% | 1.0955 | 0.2930 | 0.08 |
| 5 | 96.51% | 16.67% | 85.33% | 1.1581 | 0.2093 | 0.13 |
| 8 | 96.12% | 16.67% | 85.00% | 1.1535 | 0.2326 | 0.13 |
| 9 | 96.12% | 19.05% | 85.33% | 1.1874 | 0.2035 | 0.15 |
| 10 | 95.74% | 19.05% | 85.00% | 1.1826 | 0.2238 | 0.15 |
| 11 | 95.35% | 19.05% | 84.67% | 1.1778 | 0.2442 | 0.14 |
| 12 | 95.35% | 21.43% | 85.00% | 1.2135 | 0.2171 | 0.17 |
| 18 | 95.35% | 23.81% | 85.33% | 1.2515 | 0.1953 | 0.19 |
| 24 | 94.57% | 23.81% | 84.67% | 1.2413 | 0.2279 | 0.18 |
| 25 | 94.19% | 26.19% | 84.67% | 1.2761 | 0.2220 | 0.20 |
| 26 | 90.31% | 30.95% | 82.00% | 1.3079 | 0.3131 | 0.21 |
| 27 | 87.98% | 38.10% | 81.00% | 1.4213 | 0.3154 | 0.26 |
| 28 | 86.05% | 45.24% | 80.33% | 1.5713 | 0.3084 | 0.31 |
| 29 | 85.66% | 47.62% | 80.33% | 1.6353 | 0.3012 | 0.33 |
| 30 | 84.88% | 52.38% | 80.33% | 1.7826 | 0.2886 | 0.37 |
| 31 | 83.72% | 52.38% | 79.33% | 1.7581 | 0.3108 | 0.36 |
| 32 | 82.17% | 52.38% | 78.00% | 1.7256 | 0.3404 | 0.35 |
| 33 | 81.78% | 52.38% | 77.67% | 1.7174 | 0.3478 | 0.34 |
| 34 | 81.78% | 57.14% | 78.33% | 1.9083 | 0.3188 | 0.39 |
| 35 | 81.40% | 59.52% | 78.33% | 2.0109 | 0.3126 | 0.41 |
| 36 | 81.01% | 61.90% | 78.33% | 2.1265 | 0.3068 | 0.43 |
| 37 | 80.23% | 61.90% | 77.67% | 2.1061 | 0.3193 | 0.42 |
| 39 | 79.84% | 61.90% | 77.33% | 2.0959 | 0.3256 | 0.42 |
| 40 | 79.46% | 64.29% | 77.33% | 2.2248 | 0.3196 | 0.44 |
| 41 | 79.07% | 64.29% | 77.00% | 2.2140 | 0.3256 | 0.43 |
| 42 | 78.68% | 64.29% | 76.67% | 2.2031 | 0.3316 | 0.43 |
| 43 | 77.91% | 64.29% | 76.00% | 2.1814 | 0.3437 | 0.42 |
| 47 | 77.13% | 66.67% | 75.67% | 2.3140 | 0.3430 | 0.44 |
| 48 | 76.36% | 66.67% | 75.00% | 2.2907 | 0.3547 | 0.43 |
| 49 | 75.97% | 66.67% | 74.67% | 2.2791 | 0.3605 | 0.43 |
| 50 | 75.97% | 69.05% | 75.00% | 2.4544 | 0.3480 | 0.45 |
| 51 | 73.64% | 71.43% | 73.33% | 2.5775 | 0.3690 | 0.45 |
| 52 | 72.48% | 71.43% | 72.33% | 2.5368 | 0.3853 | 0.44 |
| 53 | 71.32% | 71.43% | 71.33% | 2.4961 | 0.4016 | 0.43 |
| 54 | 70.54% | 71.43% | 70.67% | 2.4690 | 0.4124 | 0.42 |
| 55 | 69.38% | 76.19% | 70.33% | 2.9140 | 0.4019 | 0.46 |
| **56** | **68.22%** | **78.57%** | **69.67%** | **3.1835** | **0.4045** | **0.47** |
| 57 | 67.83% | 78.57% | 69.33% | 3.1654 | 0.4094 | 0.46 |
| 58 | 65.89% | 78.57% | 67.67% | 3.0749 | 0.4341 | 0.44 |
| 59 | 63.18% | 78.57% | 65.33% | 2.9483 | 0.4686 | 0.42 |
| 60 | 62.79% | 78.57% | 65.00% | 2.9302 | 0.4736 | 0.41 |
| 61 | 60.47% | 78.57% | 63.00% | 2.8217 | 0.5032 | 0.39 |
| 62 | 55.43% | 80.95% | 59.00% | 2.9099 | 0.5506 | 0.36 |
| 63 | 53.88% | 83.33% | 58.00% | 3.2326 | 0.5535 | 0.37 |
| 64 | 51.16% | 90.48% | 56.67% | 5.3721 | 0.5398 | 0.42 |
| 65 | 50.39% | 90.48% | 56.00% | 5.2907 | 0.5483 | 0.41 |
| 66 | 49.22% | 90.48% | 55.00% | 5.1686 | 0.5612 | 0.40 |
| 67 | 48.45% | 90.48% | 54.33% | 5.0872 | 0.5698 | 0.39 |
| 68 | 47.67% | 90.48% | 53.67% | 5.0058 | 0.5783 | 0.38 |
| 69 | 46.51% | 90.48% | 52.67% | 4.8837 | 0.5912 | 0.37 |
| 70 | 46.12% | 92.86% | 52.67% | 6.4574 | 0.5802 | 0.39 |
| 71 | 44.57% | 95.24% | 51.67% | 9.3605 | 0.5820 | 0.40 |
| 72 | 43.80% | 95.24% | 51.00% | 9.1977 | 0.5901 | 0.39 |
| 73 | 43.02% | 95.24% | 50.33% | 9.0349 | 0.5983 | 0.38 |
| 74 | 41.47% | 95.24% | 49.00% | 8.7093 | 0.6145 | 0.37 |
| 75 | 40.31% | 95.24% | 48.00% | 8.4651 | 0.6267 | 0.36 |
| 76 | 38.37% | 97.62% | 46.67% | 16.1162 | 0.6313 | 0.36 |
| 77 | 36.82% | 97.62% | 45.33% | 15.4651 | 0.6472 | 0.34 |
| 79 | 35.66% | 97.62% | 44.33% | 14.9767 | 0.6591 | 0.33 |
| 80 | 34.11% | 97.62% | 43.00% | 14.3255 | 0.6750 | 0.32 |
| 81 | 32.17% | 97.62% | 41.33% | 13.5116 | 0.6948 | 0.30 |
| 82 | 31.40% | 97.62% | 40.67% | 13.1860 | 0.7028 |  |
| 83 | 29.07% | 97.62% | 38.67% | 12.2093 | 0.7266 |  |
| 84 | 28.68% | 97.62% | 38.33% | 12.0465 | 0.7306 |  |
| 85 | 27.13% | 97.62% | 37.00% | 11.3953 | 0.7465 |  |
| 86 | 24.81% | 97.62% | 35.00% | 10.4186 | 0.7703 |  |
| 87 | 23.64% | 100.00% | 34.33% | 0.7636 |  |  |
| 88 | 22.48% | 100.00% | 33.33% | 0.7752 |  |  |
| 89 | 22.09% | 100.00% | 33.00% | 0.7791 |  |  |
| 91 | 21.71% | 100.00% | 32.67% | 0.7829 |  |  |
| 93 | 20.93% | 100.00% | 32.00% | 0.7907 |  |  |
| 94 | 20.16% | 100.00% | 31.33% | 0.7984 |  |  |
| 95 | 19.77% | 100.00% | 31.00% | 0.8023 |  |  |
| 96 | 18.22% | 100.00% | 29.67% | 0.8178 |  |  |
| 97 | 16.67% | 100.00% | 28.33% | 0.8333 |  |  |
| 98 | 14.73% | 100.00% | 26.67% | 0.8527 |  |  |
| 99 | 11.24% | 100.00% | 23.67% | 0.8876 |  |  |
| 100 | 6.20% | 100.00% | 19.33% | 0.9380 |  |  |

Bold texts emphasize the chosen cut-off value.

**Supplementary Table 3.** Optimum cut-off value calculation for AI ALA score against moderate-to-critical disease.

| Cut point (>) | Sensitivity | Specificity | Accuracy | LR+ | LR- | Youden Index |
| --- | --- | --- | --- | --- | --- | --- |
| 0 | 100.00% | 0.00% | 86.00% | 1.0000 |  | 0.00 |
| **1** | **84.50%** | **73.81%** | **83.00%** | **3.2262** | **0.2101** | **0.58** |
| 2 | 76.36% | 78.57% | 76.67% | 3.5633 | 0.3009 | 0.55 |
| 3 | 70.54% | 83.33% | 72.33% | 4.2326 | 0.3535 | 0.54 |
| 4 | 66.67% | 85.71% | 69.33% | 4.6667 | 0.3889 | 0.52 |
| 5 | 62.02% | 90.48% | 66.00% | 6.5116 | 0.4198 | 0.53 |
| 6 | 59.30% | 95.24% | 64.33% | 12.4535 | 0.4273 | 0.55 |
| 7 | 57.75% | 97.62% | 63.33% | 24.2557 | 0.4328 | 0.55 |
| 8 | 55.81% | 97.62% | 61.67% | 23.4418 | 0.4526 | 0.53 |
| 9 | 53.49% | 100.00% | 60.00% | 0.4651 |  | 0.53 |
| 10 | 51.16% | 100.00% | 58.00% | 0.4884 |  | 0.51 |
| 11 | 50.00% | 100.00% | 57.00% | 0.5000 |  | 0.50 |
| 12 | 48.06% | 100.00% | 55.33% | 0.5194 |  | 0.48 |
| 13 | 47.67% | 100.00% | 55.00% | 0.5233 |  | 0.48 |
| 14 | 46.90% | 100.00% | 54.33% | 0.5310 |  | 0.47 |
| 15 | 45.74% | 100.00% | 53.33% | 0.5426 |  | 0.46 |
| 16 | 43.02% | 100.00% | 51.00% | 0.5698 |  | 0.43 |
| 17 | 41.09% | 100.00% | 49.33% | 0.5891 |  | 0.41 |
| 18 | 39.53% | 100.00% | 48.00% | 0.6047 |  | 0.40 |
| 19 | 37.60% | 100.00% | 46.33% | 0.6240 |  | 0.38 |
| 20 | 36.82% | 100.00% | 45.67% | 0.6318 |  | 0.37 |
| 21 | 34.11% | 100.00% | 43.33% | 0.6589 |  | 0.34 |
| 22 | 32.95% | 100.00% | 42.33% | 0.6705 |  | 0.33 |
| 23 | 32.56% | 100.00% | 42.00% | 0.6744 |  | 0.33 |
| 24 | 32.17% | 100.00% | 41.67% | 0.6783 |  | 0.32 |
| 25 | 30.62% | 100.00% | 40.33% | 0.6938 |  | 0.31 |
| 26 | 30.23% | 100.00% | 40.00% | 0.6977 |  | 0.30 |
| 27 | 29.84% | 100.00% | 39.67% | 0.7016 |  | 0.30 |
| 28 | 28.29% | 100.00% | 38.33% | 0.7171 |  | 0.28 |
| 29 | 27.13% | 100.00% | 37.33% | 0.7287 |  | 0.27 |
| 30 | 26.36% | 100.00% | 36.67% | 0.7364 |  | 0.26 |
| 31 | 25.58% | 100.00% | 36.00% | 0.7442 |  | 0.26 |
| 32 | 24.42% | 100.00% | 35.00% | 0.7558 |  | 0.24 |
| 33 | 23.64% | 100.00% | 34.33% | 0.7636 |  | 0.24 |
| 34 | 22.09% | 100.00% | 33.00% | 0.7791 |  | 0.22 |
| 35 | 20.93% | 100.00% | 32.00% | 0.7907 |  | 0.21 |
| 36 | 20.54% | 100.00% | 31.67% | 0.7946 |  | 0.21 |
| 37 | 18.99% | 100.00% | 30.33% | 0.8101 |  | 0.19 |
| 38 | 18.22% | 100.00% | 29.67% | 0.8178 |  | 0.18 |
| 39 | 17.44% | 100.00% | 29.00% | 0.8256 |  | 0.17 |
| 40 | 16.67% | 100.00% | 28.33% | 0.8333 |  | 0.17 |
| 41 | 14.73% | 100.00% | 26.67% | 0.8527 |  | 0.15 |
| 43 | 14.34% | 100.00% | 26.33% | 0.8566 |  | 0.14 |
| 44 | 13.18% | 100.00% | 25.33% | 0.8682 |  | 0.13 |
| 45 | 12.79% | 100.00% | 25.00% | 0.8721 |  | 0.13 |
| 46 | 11.24% | 100.00% | 23.67% | 0.8876 |  | 0.11 |
| 47 | 10.85% | 100.00% | 23.33% | 0.8915 |  | 0.11 |
| 48 | 8.91% | 100.00% | 21.67% | 0.9109 |  | 0.09 |
| 49 | 8.53% | 100.00% | 21.33% | 0.9147 |  | 0.09 |
| 50 | 7.75% | 100.00% | 20.67% | 0.9225 |  | 0.08 |
| 51 | 7.36% | 100.00% | 20.33% | 0.9264 |  | 0.07 |
| 52 | 6.59% | 100.00% | 19.67% | 0.9341 |  | 0.07 |
| 53 | 6.20% | 100.00% | 19.33% | 0.9380 |  | 0.06 |
| 54 | 5.43% | 100.00% | 18.67% | 0.9457 |  | 0.05 |
| 55 | 4.65% | 100.00% | 18.00% | 0.9535 |  | 0.05 |
| 56 | 4.26% | 100.00% | 17.67% | 0.9574 |  | 0.04 |
| 57 | 3.88% | 100.00% | 17.33% | 0.9612 |  | 0.04 |
| 58 | 2.71% | 100.00% | 16.33% | 0.9729 |  | 0.03 |
| 59 | 1.94% | 100.00% | 15.67% | 0.9806 |  | 0.02 |
| 62 | 1.55% | 100.00% | 15.33% | 0.9845 |  | 0.02 |
| 63 | 1.16% | 100.00% | 15.00% | 0.9884 |  | 0.01 |
| 68 | 0.78% | 100.00% | 14.67% | 0.9922 |  | 0.01 |
| 70 | 0.39% | 100.00% | 14.33% | 0.9961 |  | 0.00 |
| 71 | 0.00% | 100.00% | 14.00% | 1.0000 |  | 0.00 |
| 75 | 100.00% | 0.00% | 86.00% | 1.0000 |  | 0.00 |

Bold texts emphasize the chosen cut-off value.

**Supplementary Table 4.** Optimum cut-off value calculation for Brixia Scoring System score against moderate-to-critical disease.

| Cut point (>) | Sensitivity | Specificity | Accuracy | LR+ | LR- | Youden Index |
| --- | --- | --- | --- | --- | --- | --- |
| 0 | 100.00% | 0.00% | 86.00% | 1.0000 |  | 0.00 |
| 0.5 | 77.52% | 90.48% | 79.33% | 8.1395 | 0.2485 | 0.68 |
| **1** | **76.74%** | **95.24%** | **79.33%** | **16.1163** | **0.2442** | **0.72** |
| 1.5 | 69.77% | 95.24% | 73.33% | 14.6512 | 0.3174 | 0.65 |
| 2 | 67.83% | 95.24% | 71.67% | 14.2442 | 0.3378 | 0.63 |
| 2.5 | 61.24% | 95.24% | 66.00% | 12.8605 | 0.4070 | 0.56 |
| 3 | 60.47% | 95.24% | 65.33% | 12.6977 | 0.4151 | 0.56 |
| 3.5 | 55.43% | 97.62% | 61.33% | 23.2790 | 0.4566 | 0.53 |
| 4 | 53.88% | 97.62% | 60.00% | 22.6278 | 0.4725 | 0.52 |
| 4.5 | 49.61% | 97.62% | 56.33% | 20.8372 | 0.5162 | 0.47 |
| 5 | 48.06% | 97.62% | 55.00% | 20.1860 | 0.5320 | 0.46 |
| 5.5 | 43.80% | 97.62% | 51.33% | 18.3953 | 0.5757 | 0.41 |
| 6 | 43.41% | 97.62% | 51.00% | 18.2325 | 0.5797 | 0.41 |
| 6.5 | 38.37% | 100.00% | 47.00% | 0.6163 |  | 0.38 |
| 7 | 37.98% | 100.00% | 46.67% | 0.6202 |  | 0.38 |
| 7.5 | 37.21% | 100.00% | 46.00% | 0.6279 |  | 0.37 |
| 8 | 36.82% | 100.00% | 45.67% | 0.6318 |  | 0.37 |
| 8.5 | 33.72% | 100.00% | 43.00% | 0.6628 |  | 0.34 |
| 9 | 31.78% | 100.00% | 41.33% | 0.6822 |  | 0.32 |
| 9.5 | 30.23% | 100.00% | 40.00% | 0.6977 |  | 0.30 |
| 10 | 28.68% | 100.00% | 38.67% | 0.7132 |  | 0.29 |
| 10.5 | 24.03% | 100.00% | 34.67% | 0.7597 |  | 0.24 |
| 11 | 22.09% | 100.00% | 33.00% | 0.7791 |  | 0.22 |
| 11.5 | 17.83% | 100.00% | 29.33% | 0.8217 |  | 0.18 |
| 12 | 16.28% | 100.00% | 28.00% | 0.8372 |  | 0.16 |
| 12.5 | 14.73% | 100.00% | 26.67% | 0.8527 |  | 0.15 |
| 13 | 12.40% | 100.00% | 24.67% | 0.8760 |  | 0.12 |
| 13.5 | 10.85% | 100.00% | 23.33% | 0.8915 |  | 0.11 |
| 14 | 9.30% | 100.00% | 22.00% | 0.9070 |  | 0.09 |
| 14.5 | 7.36% | 100.00% | 20.33% | 0.9264 |  | 0.07 |
| 15 | 6.59% | 100.00% | 19.67% | 0.9341 |  | 0.07 |
| 15.5 | 6.20% | 100.00% | 19.33% | 0.9380 |  | 0.06 |
| 16 | 4.26% | 100.00% | 17.67% | 0.9574 |  | 0.04 |
| 16.5 | 3.10% | 100.00% | 16.67% | 0.9690 |  | 0.03 |
| 17 | 2.71% | 100.00% | 16.33% | 0.9729 |  | 0.03 |
| 17.5 | 1.94% | 100.00% | 15.67% | 0.9806 |  | 0.02 |
| 18 | 0.78% | 100.00% | 14.67% | 0.9922 |  | 0.01 |

Bold texts emphasize the chosen cut-off value.

**Supplementary Figure 1.** Interobserver consistency of Brixia scoring system based on CXR projections presented with ICC coefficient and 95%CI.


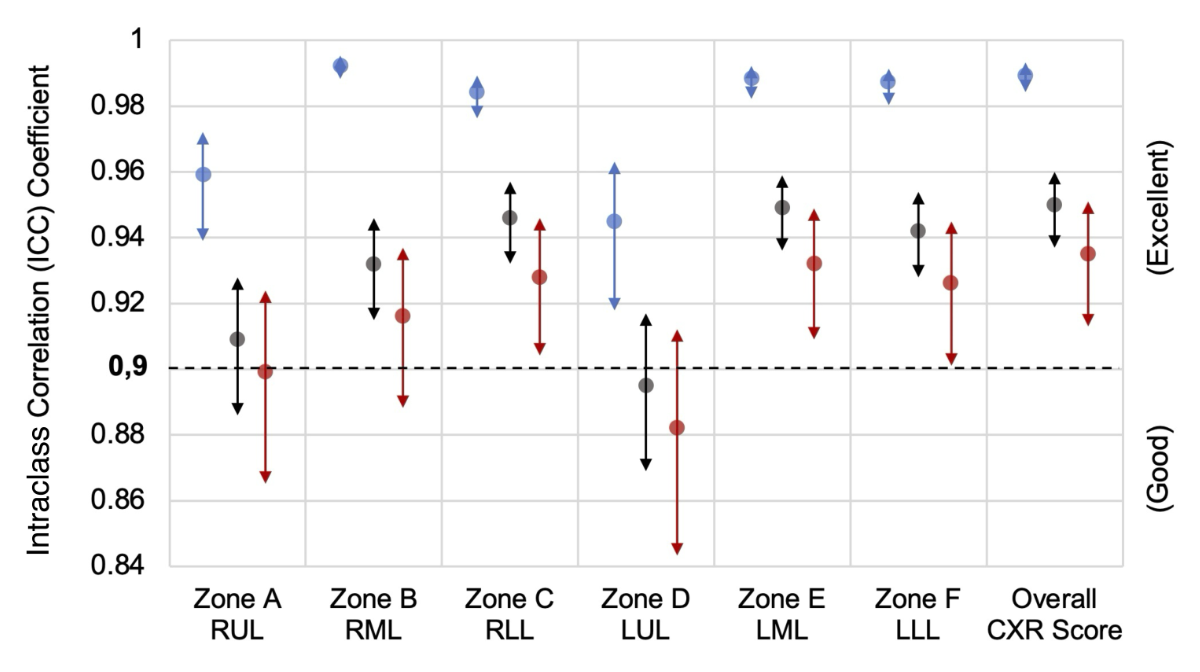


_⚫️,_ Total; _🔵,_ Postero-Anterior; _🔴,_ Antero-Posterior; RUL, Right Upper Lobe; RML, Right Middle Lobe; RLL, Right Lower Lobe; LUL, Left Upper Lobe; LML, Left Middle Lobe; LLL, Left Lower Lobe; CXR, Chest X-Ray. Intraclass correlation coefficient value: 0.75-0.90, good agreement; > 0.90, excellent agreement.

**Supplementary Figure 2.** Distribution of Binarized AI scores and Brixia Scoring System score.

**
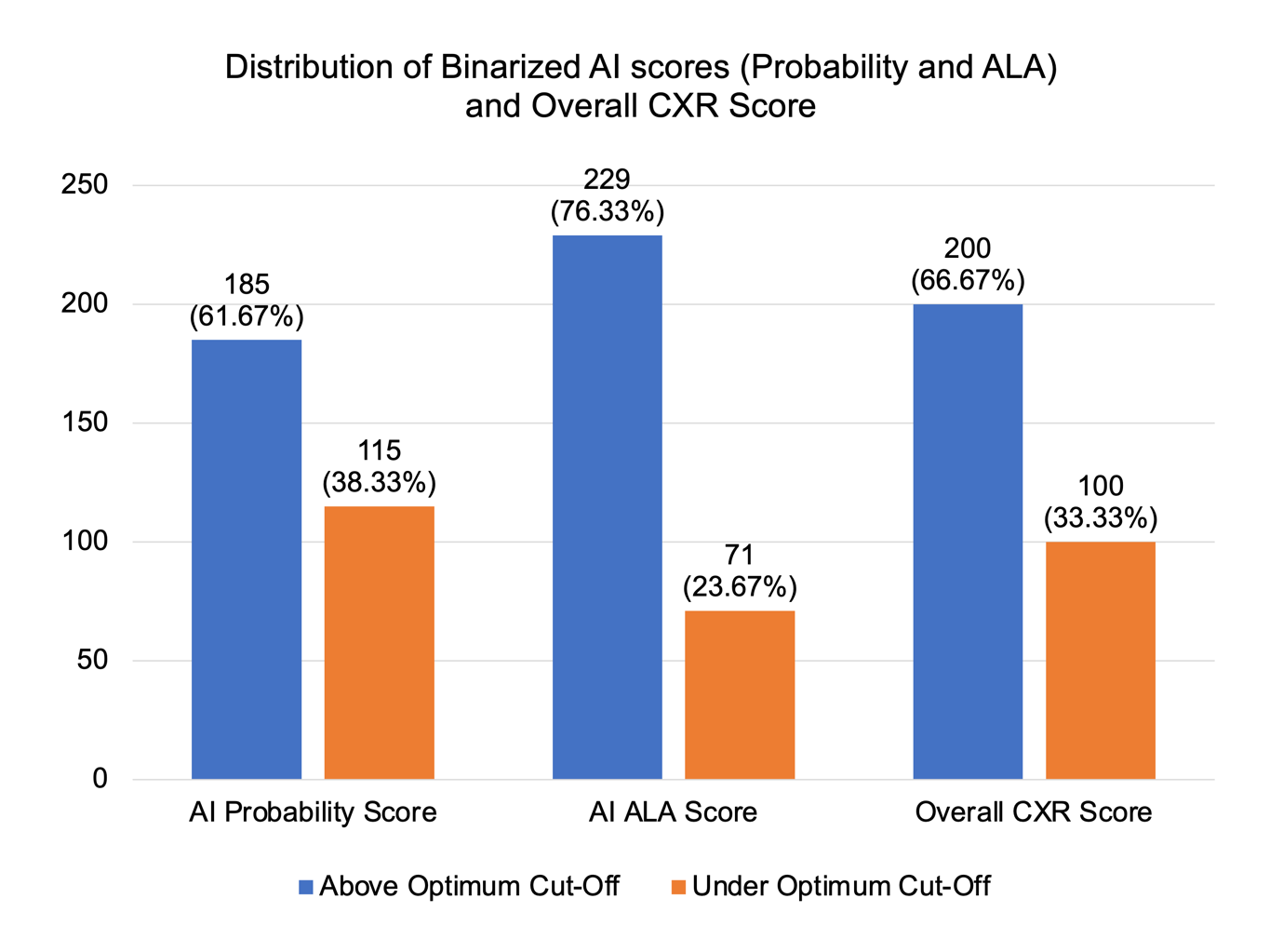
**
